# Dynamic Regulation of Vascular Myosin Light Chain (MYL9) with Injury and Aging

**DOI:** 10.1371/journal.pone.0025855

**Published:** 2011-10-07

**Authors:** Lina A. Shehadeh, Keith A. Webster, Joshua M. Hare, Roberto I. Vazquez-Padron

**Affiliations:** 1 Department of Medicine, Division of Cardiology, University of Miami Leonard M. Miller School of Medicine, Miami, Florida, United States of America; 2 Vascular Biology Institute, University of Miami Leonard M. Miller School of Medicine, Miami, Florida, United States of America; 3 Interdisciplinary Stem Cell Institute, University of Miami Leonard M. Miller School of Medicine, Miami, Florida, United States of America; 4 Department of Pharmacology, University of Miami Leonard M. Miller School of Medicine, Miami, Florida, United States of America; 5 Department of Surgery, University of Miami Leonard M. Miller School of Medicine, Miami, Florida, United States of America; Brigham & Women's Hospital - Harvard Medical School, United States of America

## Abstract

**Background:**

Aging-associated changes in the cardiovascular system increase the risk for disease development and lead to profound alterations in vascular reactivity and stiffness. Elucidating the molecular response of arteries to injury and age will help understand the exaggerated remodeling of aging vessels.

**Methodology/Principal Findings:**

We studied the gene expression profile in a model of mechanical vascular injury in the iliac artery of aging (22 months old) and young rats (4 months old). We investigated aging-related variations in gene expression at 30 min, 3 d and 7 d post injury. We found that the Myosin Light Chain gene (**MYL9**) was the only gene differentially expressed in the aged versus young injured arteries at all time points studied, peaking at day 3 after injury (4.6 fold upregulation (*p<0.05*) in the smooth muscle cell layers. We confirmed this finding on an aging aortic microarray experiment available through NCBI's GEO database. We found that Myl9 was consistently upregulated with age in healthy rat aortas. To determine the arterial localization of Myl9 with age and injury, we performed immunohistochemistry for Myl9 in rat iliac arteries and found that in healthy and injured (30 days post injury) arteries, Myl9 expression increased with age in the endothelial layers.

**Conclusions/Significance:**

The consistent upregulation of the myosin light chain protein (Myl9) with age and injury in arterial tissue draws attention to the increased vascular permeability and to the age-caused predisposition to arterial constriction after balloon angioplasty.

## Introduction

Vascular diseases remain the most common cause of death in the world [1]. Aging increases the risk for hypertension, coronary artery disease, and heart failure. The aging-associated changes in the cardiovascular system lead to profound alterations in vascular reactivity and stiffness [2], [3]. Aging also increases the risk for vascular proliferative diseases such atherosclerosis [4] and restenosis after percutaneous coronary interventions.

In fact, experimental data suggesting that age increases the risk for vascular diseases date back to 1982. Using a balloon injury model Stemerman et al. [5] showed that VSMCs of aged rats proliferate more in response to injury than those of young animals. These results were reproduced in rabbits and primates [4], [6] and extended to a model of vascular injury in mice [7]. We previously demonstrated that aged mice develop more neointima following arterial injury than their younger counterparts [7].

Based on the aforementioned observations, the aim of this study was to investigate the effects of aging on the early vascular response to injury in a model of arterial stenosis. Therefore, we studied the global gene expression profiles in healthy and injured arteries of aged and young rats at multiple time points. Moreover, we attempted to verify our results by analysis of a public dataset on gene expression of aged and young healthy aortas spanning four different ages [8]. Finally, we studied the localization of the identified contractility gene, Myl9, within the arterial wall of aged and young, healthy and injured iliac arteries.

## Results

### Myl9 is the only gene differentially expressed in all 3 different time-points after wire injury in aged versus young rats

**Figure 1:**
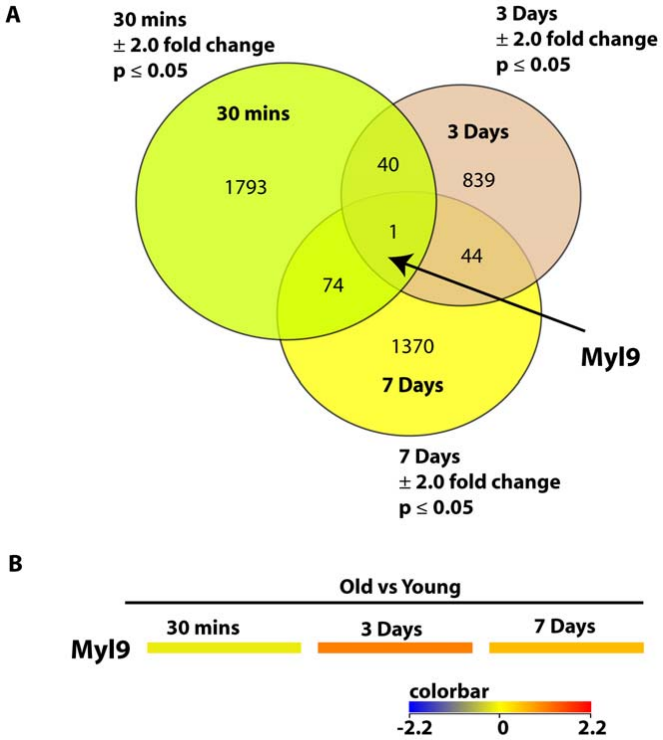
Differentially expressed genes overlapping in multiple time points in old versus young injured rat iliac arteries. A. Venn diagram shows the number of differentially expressed genes determined by our analysis of 3 different time points in old versus young injured rat iliac arteries. Gene lists were compared to find common differentially expressed transcripts. A 2.0 fold change and p<.05 significance cut-offs were used. No multiple correction was employed. B. Heat map of Myl9 gene expression levels in multiple time points in old versus young injured rat iliac arteries. Myosin Light chain 9, Myl9, was significantly downregulated at 30 mins (−2.2 fold), highly upregulated at 3 days (4.6 fold), and upregulated at 7 days (2.9 fold). Color bar shown in Log_2_.

We can start to comprehend the dynamics of vascular injury only if we examine vascular remodeling with time. Therefore, we elected to study global gene expression of vascular remodeling in a 3-point time series. Looking at the global gene expression in the iliac arteries of old versus young rats 30 minutes, 3 days, or 7 days after balloon injury, we found that Myosin Light Chain, Myl9, was the only gene that overlapped as differentially expressed in all the three experiments (Figure 1). Myl9, was significantly downregulated at 30 mins (−2.2 fold), highly upregulated at 3 days (4.6 fold), and upregulated at 7 days (2.9 fold). 40 other genes overlapped between 30 minutes and 3-days; 44 other genes overlapped between 3-day and 7-day injury; 74 genes overlapped between 3 minutes and 7-day injury. A comprehensive list of the overlapping genes is provided in Supplemental Table S1.A 2.0 fold change and p<.05 significance cut-offs were used. No multiple correction was employed.

### Age has a profound effect on genes differentially expressed after 30 minutes of wire injury in rats

**Figure 2:**
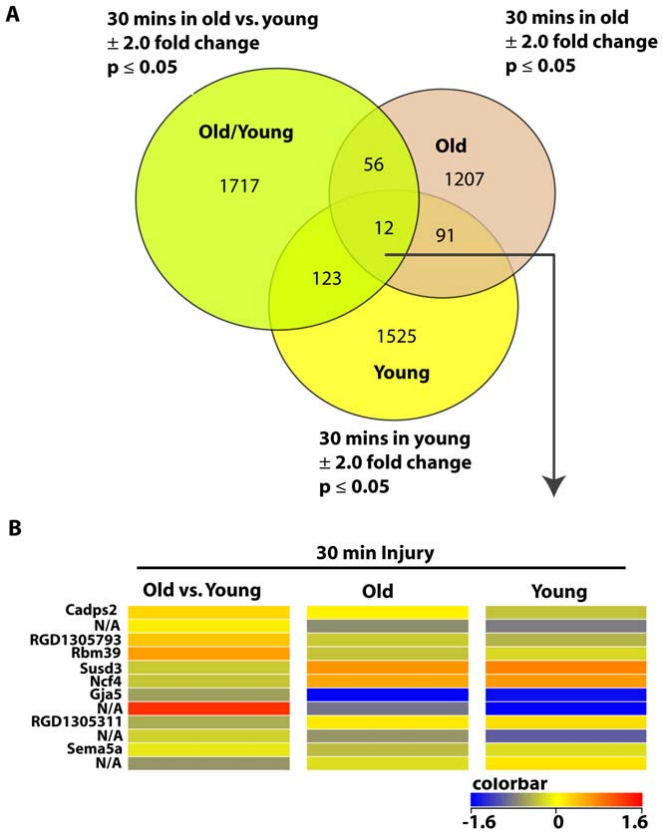
Differentially expressed genes overlapping in different age groups of 30 min-injured rat iliac arteries. A. Venn diagram shows lists of differentially expressed genes determined by our analysis of 3 different pairs of age groups of 30 min-injured rat iliac arteries. Gene lists were compared to find common differentially expressed transcripts. A 2.0 fold change and p<.05 significance cut-offs were used. No multiple correction was employed. B. Heat map of differentially expressed genes overlapping in different age groups of 30 min-injured rat iliac arteries.12 genes overlapped in old versus young, old versus old, and young versus young, 30 min-injured rat iliac arteries. Color bar shown in Log_2_.

Vascular remodeling is highly sensitive to age. Therefore, to examine the effect of age on vascular remodeling using a common 30 minutes post-injury time, we studied the global gene expression in the iliac arteries of old versus young, old versus old, and young versus young rats. While most of the genes did not overlap among the 3 pairs compared, 12 genes overlapped in all the 3 experiments (Figure 2AB). These 12 genes can be considered not sensitive to the age effect but rather unique to the injury process. They include the Ca2+-dependent activator protein (Cadps2), the RNA binding motif protein (Rbm39), the neutrophil cytosolic factor (Ncf4), and the gap junction protein (Gja5). A comprehensive list of the overlapping genes between any 2 conditions is provided in Supplemental Table S2.A 2.0 fold change and p<.05 significance cut-offs were used. No multiple correction was employed.

### A common aging transcriptional profile in healthy rat iliac and aortic arteries includes Myl9

**Figure 3:**
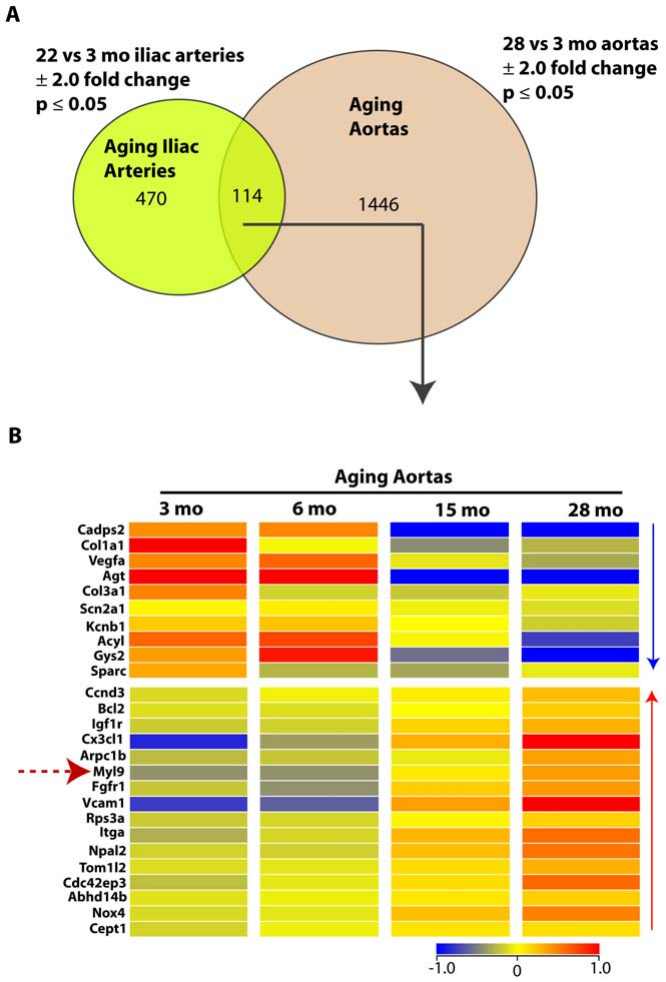
Differentially expressed genes overlapping in healthy aging rat iliac arteries and aortas. A. Venn diagram shows differentially expressed genes determined by our analysis of healthy aging rat iliac arteries and healthy aging rat thoracic aortas. The two gene lists were compared to find common differentially expressed transcripts. A 2.0 fold change and p<.05 significance cut-offs were used. No multiple correction was employed. B. Heat map of differentially expressed genes overlapping in healthy aging rat iliac arteries and aortas. Selected genes, including Myl9, from the 114 transcripts overlapping in healthy aging iliac arteries and thoracic aortas, are shown in a heatmap displaying their expression levels in young and old aortas. Color bar shown in Log_2_.

To investigate for a common aging transcriptional profile in healthy vessels, we compared global gene expression between 1) aged (22 months) versus young (3 months) rat iliac arteries, and 2) aged (28 months) versus young (3 months) rat thoracic aortas [8]. We found that 114 transcripts overlapped in the two studies (Figure 3A). Among these common transcripts were the contraction genes Myl9 (Figure 3B) and Itga1 (Integrin alpha 1), which increased systematically with age in rat thoracic aortas (Figure 3B). As expected, collagens (Col1a1 and Col3a1) and vascular endothelial growth factor (Vegfa) were consistently downregulated, and the aging genes B-cell leukemia/lymphoma 2 (Bcl2) and vascular cell adhesion molecule 1 (Vcam1) were upregulated in both iliac and aortic arteries with age. In addition, NADPH oxidase 4 (Nox4) which is a positive regulator of SMC migration was also upregulated with age. A 2.0 fold change and p<.05 significance cut-offs were used. No multiple correction was employed.

### Myl9 protein in over-expressed in aged versus young iliac arteries and is concentrated in the endothelial layer

**Figure 4:**
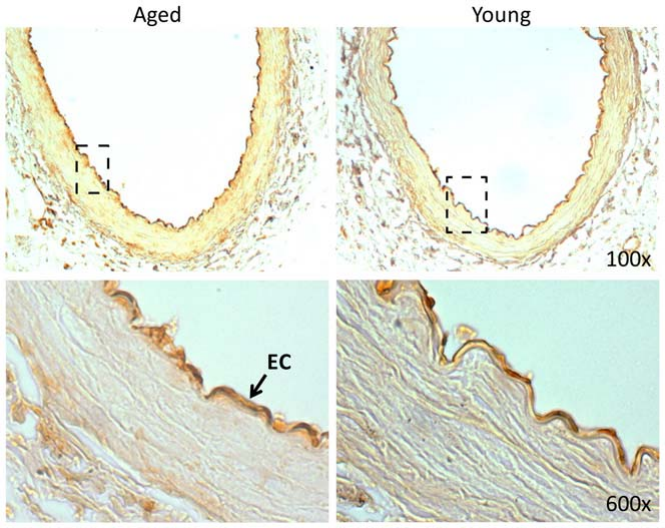
Myl9 immunostaining of old (22 months) and young (3 months) non-injured rat iliac arteries. Shown are representative images of young and old healthy iliac arteries immunostained for Myl9. Results confirm over-expression of Myl9 in the older group and point to concentration of Myl9 expression in the endothelial layer of the iliac arteries.

**Figure 5:**
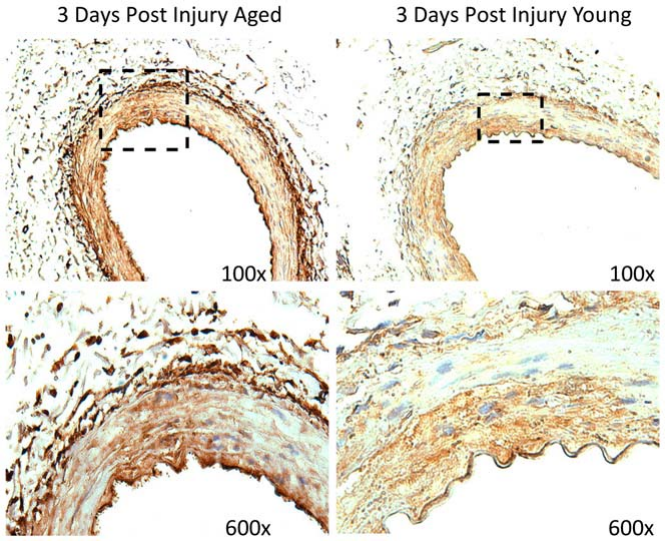
Myl9 immunostaining of old (22 months) and young (3 months) injured rat iliac arteries. Shown are representative images of young and old iliac arteries 3 days post injury, immunostained for Myl9. Results confirm over-expression of Myl9 in the older injured group and point to concentration of Myl9 expression in the smooth muscle layer of the iliac arteries during this post injury transition period.

**Figure 6:**
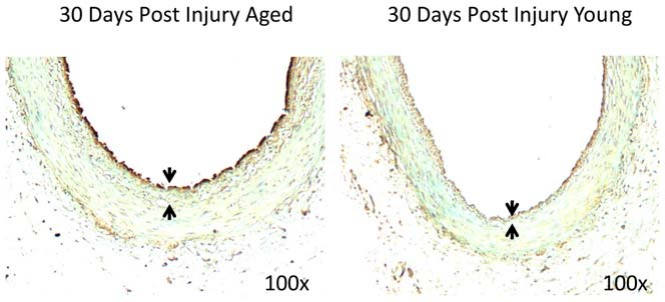
Myl9 immunostatining of old (22 months) and young (3 months) injured rat iliac arteries. Shown are representative images of young and old iliac arteries 30 days post injury, immunostained for Myl9. Results confirm over-expression of Myl9 in the older injured group and point to concentration of Myl9 expression in the endothelial layer of the iliac arteries during this post injury period.

To confirm the over-expression of Myl9 in aged versus young arteries, we performed immunohistochemical staining for Myl9 in young (3 months) and older (22 months) healthy rat iliac arteries. Results confirm over-expression of Myl9 in the older group and point to concentration of Myl9 protein expression in the endothelial layer of the healthy iliac arteries (Figure 4). Arterial injury also induced Myl9 expression with age. Interestingly, the localization of Myl9 was dependent on the post-injury time. Myl9 was highly over-expressed in the smooth muscle cell layer of the injured arteries 3 days post insult (Figure 5), whereas Myl9 was concentrated in the endothelial layer 30 days post injury (Figure 6).

## Discussion

The present findings show that vascular remodeling is a dynamic process that is greatly influenced by age and post-injury time. While we found that the transcriptional programs clearly vary among age groups and post-injury times, we identified a common denominator for aging healthy arteries and aging injured arteries at various post-injury times. The contractile regulatory gene myosin light chain (Myl9) was consistently upregulated in healthy aging aortas and was the only differentially expressed gene in injured iliac arteries at all 3 post-injury time points explored (30 minutes, 3 days, and 7days).

Myosins are a diverse superfamily of actin-dependent molecular motors consisting of distinct structural and functional classes and are implicated in contraction, cell shape, migration, adhesion, intracellular transport of organelles, and signal transduction [9]. Conventional myosin II-complexes are hexamers consisting of 2 heavy chains, 2 regulatory MLCs, and 2 essential light chains. The activity of myosin is regulated by reversible phosphorylation of specific amino acids in the regulatory MLC [9]. In the vascular muscle, the interaction of myosin with actin is the primary determinant of force production (contraction). Arterial vascular smooth muscle cells contain abundant amounts of smooth muscle myosin heavy chain (gene MYH11) that are generated by alternative splicing of exons in the head (SM-A,B) and tail (SM1,2) of the motor protein [10].The consequences of the differential distribution of muscle and non- muscle MLC isoforms in vascular tissues are unknown though it has been proposed that MLCs influence myosin ATPase activity and velocity of shortening [11]. We found that Myl9 expression transiently increased with age in the smooth muscle cell layers of injured arteries (3 d post-injury). Our finding is in agreement with previously published work demonstrating that aging rats showed an increased vascular negative remodeling (constriction) after balloon dilatation compared with adult animals[12]. This agrees with the aggressive circumferential coronary constriction (‘recoil’) observed after balloon angioplasty in patients with coronary artery diseases and animal models [12], [13]. Our observation is also in accordance with previous findings from the literature reporting that the effect of phosphorylation and de-phosphorylation of Myl9 protein to be at the essence of altered vascular contractility [14].

Interestingly, we found that in healthy and late injured (30 days post injury) iliac arteries, Myl9 expression increased with age in the endothelial layers. Our findings are in line with those recently published by Licht et al [15], describing Jun-b dependent expression of Myl9 in primary cultures of endothelial cells (ECs). The effect of contractility proteins on aged-related vascular remodeling and their localization in the EC versus SMC layers is not well understood. Zeng et al had also found Myl9 expressed in ECs, which upon phosphorylation regulated endothelial cytoskeletal remodeling, and increased endothelial cell contraction resulting in increased paracellular gap formation and endothelial hyperpermeability [16]. If Myl9 ectopic expression causes endothelial dysfunction, it may explain the increased signs of arterial constriction in the aging vasculature. For instance, age-related endothelial dysfunction in aged rat aortas was found associated with decreased vasorelaxation likely caused by a decline in NO and endothelium-derived hyperpolarizing factor [17], [18].

Myosin light chain phosphorylation may play an important role in endothelial function, including reorganization of the cytoskeleton, changing cell shape, and controlling endothelial cell isometric tension [19], [20]. Interestingly, endothelial permeability appears quite early in the progress of aging. In fact aortas of 30-month-old rats had a 2-fold increase in endothelial permeability to albumin compared with 10-month-old rats [21]. Endothelial cells lining blood vessels form a continuous layer that constrains proteins and blood elements to the vascular lumen. An increase in endothelial cell isometric tension (contraction) may disrupt the continuous endothelial barrier leading to an increase in permeability and development of edema, a hallmark of acute and chronic inflammation. It is believed that elevated permeability of the endothelium allows entry of lipoproteins into the vessel wall, which become oxidized and propagate endothelial dysfunction [22]. The relevance of vascular permeability in the development of age-related vascular diseases have been extensively discussed [23], [24]. Of note, endothelial leakage favors the passage of plasma macromolecules across the endothelium and their trapping in the intima, which could contribute to the development of restenosis and atherosclerosis. The increased endothelial Myl9 may also explain the morphological changes of endothelial cells associated with aging which could account for the altered endothelial permeability [25]. Conversely, phosphorylation of myosin regulatory light chain (MLC) not only increases actomyosin ATPase activity, but also destabilizes the endothelial cell−cell junctions leading to increased monolayer cell permeability [26], [16], [27].Our results are encouraging and they warrant future research on demonstrating whether ectopic expression of Myl9 in the aged endothelium causes endothelial dysfunction.

In conclusion, our findings suggest that the amount and localization of Myl9 is critical in the vascular function and remodeling process with age. The analysis presented here sets the basis for future studies in the field of vascular aging, in particular for those aimed at preventing age-related vascular dysfunction. Based on the knowledge of the role of Myl9 in endothelium degeneration, strategies to prevent this process could be designed and tested. New therapeutic interventions to prevent vascular aging might have enormous medical consequences given the strong age dependency of cardiovascular diseases.

## Methods

### Rat Balloon Injury Model

Aged Fisher (>22-month-old, F344) rats were purchased from the National Institute of Aging (Bethesda, MD). Young (2-month-old) rats were obtained from Harlan Laboratories (Indianapolis, IN). Animal work was revised and approved by the Institutional Committee for Use and Care of Laboratory Animals at the University of Miami protocol approval ID 05-069 entitled “The role of vascular senescence in age-related vasculopathies”. All operative procedures were under isoflurane anesthesia (Baxter, IL, USA). Balloon injury in the right iliac artery was inflicted with a 2F Fogarty catheter (Baxter Corp., Irvine, CA, USA) adapted to a custom angiographic kit (Boston Scientific, Scimed) [28]. The balloon catheter was always inflated to yield a constant pressure between 1.5–1.6 atmospheres. Arterial specimens were collected 30 min, 3, 7 and 30 days after injury, cut in two fragments that were fixed in 4% formalin-PBS or submerged in RNA later until RNA isolation. All animal procedures were previously approved by the Institutional Committee for Use and Care of Laboratory Animals at the University of Miami.

### RNA Isolation for Gene Arrays

To isolate total RNA from the each of the 40 iliac artery samples, 0.3 mL of TRI Reagent (Molecular Research Center Cat #TRI-118) was added per tissue sample. The sample was then macerated using a Kinematica AG Polytron PT-2100 for 1 min. After a 5 min incubation at room temperature 0.2 volumes of chloroform was added and mixed, following by a 3 min incubation at room temperature and 10 min centrifugation at 12,000 g 4°C. The top aqueous layer was transferred to a new tube containing 0.5 volume of isopropanol and 2 to 10 ul of polyacryl carrier (Molecular Research Center, Cat # PC152). After mixing, the precipitate containing the total RNA was collected by 15 min of centrifugation at 12,000 g 4°C. The pellet was then washed two times with 70% ethanol. Total RNA was additionally purified with RNeasy Mini Kit (Qiagen, Cat # 74106). Total RNA yield was determined spectrophotometrically.

### RNA Qualification by Agilent

To characterize the quality of an RNA sample, it was analyzed on the Agilent 2100 Bioanalyzer. Briefly, approximately 100 to 200 ng of the sample was placed in an RNA LabChip with the appropriate sample loading buffer and electrophoresed in parallel with an RNA standard ladder (Ambion cat # 7152). Agilent Technologies’ Bioanalyzer software was used to analyze the results and to create an electropherogram. RNA was considered usable if ratio of 28S ribosomal peak to 18 S ribosomal peak was above 1.0. All the 40 RNA samples used in our expression study had RIN values between 6.4 and 7.2.

### Labeling and Hybridization

10 to 20 ug of total RNA were mixed with the 200 pMol of oligo-dT primer. After 10 min incubation at 70°C and 5 min at 4°C, 20 units of Transcriptor reverse transcriptase (R°C he, Cat # 3531287001), 10 nMol of each dNTP and 4 nMol of aminoallyl-dUTP (Ambion, Cat # 8439) were added, and this mix was incubated 2 hours at 42°C. After 30 min RNase treatment cDNA was purified using QIAquick PCR purification Kit (Qiagen, Cat # 28106). Amount of cDNA was determined spectrophotometrically, and samples were dried on speedvac. cDNA was resuspended in the carbonate buffer (pH 9.0−9.3) and mixed with the Cy3- and Cy5-NHS ethers (Amersham, Cat # PA23001 and PA25001, respectively). After 1 hour incubation in the dark 20 mMol of hydroxylamine were added to quench the reaction, and labeled cDNA was purified using QIAquick PCR purification Kit. Concentration of the labeled cDNA and labeling efficiency were determined spectrophotometrically, and labeled cDNA were hybridized to Agilent Whole Rat Genome Arrayfor 17 hours at 60°C according to manufacturer’s instructions. Two samples were hybridized per chip. Therefore, the 40 samples were run on 20 arrays.

### Image Analysis and Data Processing

The microarrays were scanned at 5 micron resolution using a GenePix4000B scanner (Axon Instruments at Molecular Devices) and the resulting images were analyzed with the software package GenePixPro 6.0 (Axon Instruments at Molecular Devices). Data extracted from the images were transferred to the software package Acuity 4.0 (AxonInstruments) for normalization and statistical analysis. Each array was normalized for signal intensities across the whole array and locally, using Lowess normalization. Features for further analysis were selected according to the following quality criteria: (1) at least 90% of the pixels in the spot had intensity higher than background plus two standard deviations; (2), there were less than 2% saturated pixels in the spot; (3) signal to noise ratio (defined as ratio of the background subtracted mean pixel intensity to standard deviation of background) was 3 or above for each channel; (4) the spot diameter was between 110 and 150 micron; (5)the regression coefficient of ratios of pixel intensity was 0.6 or above.

All data is MIAME compliant and all raw .gpr files from the 20 arrays are deposited in NCBI’s GEO database (GSE29255), a MIAMI compliant database.

### Gene Arrays: Global Transcription Analysis

All 20 raw gpr files (corresponding to the 40 samples of iliac arteries) were imported into GeneSpring GX 11 software (Silicon Genetics, Redwood City, CA). Each condition had 3−4 replicates/arrays. Normalized expression values were calculated by the Robust Multi-array Average (RMA) method. The resultant signal information was analyzed using one-way analysis of variance (ANOVA) (*p<0.05*), assuming normality and equal variances. No correction for multiple comparisons was done. GeneSpring’s cross gene error model, which determines the likelihood of observing a specific fold change to the likelihood of observing a fold measurement by the 50.0 th percentile of all measurements in that sample, then setting the average value of expression level for each gene across the samples to 1.0, and plotting the resulting normalized signal value for each sample (values below 0.01 were set to 0.01). The list and the order of various genes in which they appear in the heatmaps can be viewed in tabular form in the supplemental files.

### Analysis of Previous Aging Aortas Microarray Datasets

Similarly, we performed extensive analysis on a publicly available microarray experiments on aging rat thoracic aortas: (A) Sixteen chips from the rat thoracic aorta tissue from 3, 6, 18, and 28 month old rats (n = 4 per group) [8] (GEO Accession: GSE7281). All 16 raw expression files were normalized using RMA processor. All normalized expression data was analyzed using GeneSpring software. Following normalization one-way analysis of variance was performed for each gene to identify statistically significant gene expression changes. Two criteria were used to determine whether a gene was differentially expressed: fold change of ±2.0 and p value <.05 using a two-tailed distribution. No correction for multiple comparisons was done. Lists of differentially expressed genes from different experiments were compared within GeneSpring and displayed as Venn diagrams to show overlapping and non-overlapping genes (Figure 3). As in our previous work [29], the convergence of differentially expressed genes (Venn diagrams) is what was used to replace the multiple testing in further screening the genes that passed the <.05 significance levels.

### Immunohistochemistry

Cross sections were taken from different levels of the paraffin-embedded arteries. After tissue rehydration, endogenous peroxidase was blocked with 3% hydrogen peroxide. Epitope retrieval was performed with citrate buffer (10 mM sodium citrate, pH 6.0) in a Pascal chamber. Non-specific binding was blocked with 0.5% blocking solution (DAKO, Carpinteria, CA). Slides with the Myl9 primary antibody (1∶250, Abcamab64161)were incubated for 1 hour at room temperature (RT). Biotinylated secondary antibodies (DAKO Universal link) were applied for 30 minutes, followed by a washing step with PBS and 15 min incubation with HRP-streptavidin solution (DAKO) at RT. Color was developed with a DAB chromogenic solution (DAKO). Nuclei were counterstained with Meyer’s hematoxylin and mounted as described above. Images were taken with an Olympus 1X71 camera fitted to an Olympus BX 40 microscope (Olympus America Inc, Center Valley, PA).

## Supporting Information

Table S1 **B: Differentially Expressed Genes in Old Injured Versus Young Control Iliac Arteries at 3 Days Post-Injury.** Listed are the 924 total transcripts differentially expressed by 2 fold (p<.05 with no multiple correction) in old Injured versus young control iliac arteries at 3 days post injury (n = 3 per group).
(XLS)

Table S2 **A: Differentially Expressed Genes in Young Injured Versus Young Control Iliac Arteries at 30 Minutes Post-Injury.** Listed are the 1751 total transcripts differentially expressed by 2 fold (p<.05 with no multiple correction) in young Injured versus control iliac arteries at 30 minutes post injury (n = 3 per group).
(XLS)
